# The health-related quality of life for hemiarthroplasty and total hip arthroplasty in the elderly: A meta-analysis

**DOI:** 10.3389/fmed.2023.1022584

**Published:** 2023-02-24

**Authors:** Yaping Su, Ruiling Li, Xiangying Ren, Yuanyuan Wang, Xiaolu Bai, Yurui Zhang, Lingyu Han, Yiman Wang, Ran Liang

**Affiliations:** ^1^School of Nursing and Health, Henan University, Kaifeng, China; ^2^Department of Evidence-Based Medicine, Zhongnan Hospital of Wuhan University, Wuhan, China; ^3^Department of Continuing Education, The First Associated Hospital of Henan University, Kaifeng, China; ^4^Department of Respiratory and Critical Care Medicine, Huaihe Hospital of Henan University, Kaifeng, China

**Keywords:** elderly, femoral neck fracture, hemiarthroplasty, total hip arthroplasty, the health-related quality of life, EU-5Q, perioperative nursing

## Abstract

**Purpose:**

What constitutes the optimal surgical plan for femoral neck fractures (FNFs) in elderly patients is controversial. The European quality of life 5-Dimension Questionnaire (EU-5Q) is an international scale used to measure the health-related quality of life (HRQoL) after surgery. We aim to verify the hip arthroplasty effect in elderly patients by analyzing HRQoL scores in the EU-5Q scale.

**Methods:**

We searched the EBSCO, Embase, PubMed, Ovid, Cochrane Library, and Web of Science databases using strict searching from established to 30 November 2022; used the Cochrane Library's Risk of Bias Assessment Tool and the Newcastle–Ottawa Scale to evaluate the literature; and used RevMan5.4.1 software to perform a meta-analysis. All the included studies used the EU-5Q scale to validate the overall outcomes for elderly hip arthroplasty.

**Results:**

The final included literature is composed of four RCTs, two cohort studies, three case–control trials, and three cross-sectional surveys. This study compared HRQoL scores measured by the EU-5Q scale, including 328 elderly patients with total hip arthroplasty (THA) and 323 elderly patients with hemiarthroplasty, which is statistically significant (OR = 0.05; 95% CI, 0.02~0.08; *P* = 0.002). The subgroups were as follows: unipolar vs. bipolar and cemented vs. uncemented hemiarthroplasty (OR = 0.06; 95% CI, 0.03~0.08; *P* < 0.001), follow-up time and age arthroplasty (OR = 0.16; 95% CI, 0.11~0.22; *P* < 0.001), molecular exercise and enhanced recovery after surgery (ERAS) (OR = 0.02; 95% CI,−0.03~0.07; *P* = 0.38), and analysis of hemiarthroplasty with cognitive dysfunction vs. the normal group (OR = 0.17; 95% CI, 0.08~0.26; *P* < 0.001). The outcome analysis was consistent with the included studies, and HRQoL of the EU-5Q scale is sensitive to surgical outcomes between THA and hemiarthroplasty.

**Conclusion:**

Surgeons still need to further evaluate and verify whether the hip arthroplasty surgical program or effect in elderly patients is optimal. Hemiarthroplasty operations in elderly patients have pointed toward a new direction for clinical treatment, and HRQoL scores measured by the EU-5Q can sensitively reflect the rehabilitation status after hip arthroplasty surgery. Moreover, the extensive correlation between surgical outcomes and perioperative neurocognitive function should be further investigated.

## Introduction

The number of femoral neck fractures (FNFs) in the elderly population has been increasing drastically in the last few decades, and it will be 6.3 million by 2050 (1). The growing number of FNFs places a heavy burden on the health-related quality of life (HRQoL) of the global healthcare system (2). As the number of elderly patients with FNFs increases with the global aging trend, this study aims to analyze the differences in HRQoL scores between posterior femoral neck surgery in elderly patients, aiming to provide a basis for selecting optimal surgically effective nursing interventions. In order to improve the mobility and satisfaction of patients with FNFs, we should make great efforts in perioperative training and management (1, 3). In fact, the activity of daily living (ADL) and HRQoL, as well as mobility, pain, mortality, and neurocognitive function are more closely related to the surgical effect (4). The results of five RCTs of THA vs. hemiarthroplasty in elderly patients showed that postoperative dislocation of THA was higher than in the hemiarthroplasty group, and during the 1 and 2-year follow-up time, the incidence of THA reoperation was also higher, but the incidence of other complications such as pneumonia, hematoma, arrhythmia, congestive heart failure, deep vein thrombosis was lower than hemiarthroplasty (4). Consequently, we analyzed the applicable literature on HRQoL in elderly patients with THA and hemiarthroplasty, aiming to improve hip arthroplasty rehabilitation in elderly patients with FNFs.

The European quality of life 5-Dimension Questionnaire (EU-5Q) scale is responsive to the changes in HRQoL scores, including persons with cognitive impairment, and the EU-5Q has advantages in the setting items for postoperative follow-up (5, 6). It includes a visual analog pain assessment tool and an HRQoL assessment tool with five dimensions, namely, mobility, self-care, activities of daily living, pain or discomfort, and anxiety or depression (7). It is widely used and has been proven to have good reliability, validity, and sensitivity in a variety of populations (8).

In recent years, research on functional reporting and outcome variables of elderly hip arthroplasty surgery has become more and more popular, for example, the enhanced recovery after surgery (ERAS) protocols, which involved surgeons, nurses, dietitians, etc., are designed to facilitate the speed of recovery, decrease the patients cost, improve the medical quality, etc. (9). Then, there are many imaging and functional outcome assessment methods and indicators for the prognosis, but there are few articles about the HRQoL evaluation of EU-5Q for elderly hip arthroplasty. The future of medical science is not only to treat diseases but also to pay more attention to the patient's overall health (10). The EU-5Q questionnaire is an international evaluation tool to effectively evaluate the surgery outcome. It provides the best treatment plan and nursing measures for elderly patients with FNFs by performing the positive reflection of the surgical effect measured by the EU-5Q scale. It uses the HRQoL score of elderly hip arthroplasty as the outcome index. First, it reflects the comparison of THA and hemiarthroplasty surgery. Second, it reflects that the EU-5Q scale can effectively measure the HRQoL status after hip arthroplasty in elderly patients. Moreover, in future research, from the comparison of the HRQoL scores, we can find out the health deficiencies of these elderly patients, which is convenient for clinical symptomatic treatment and nursing.

## Materials and methods

### Inclusion and exclusion criteria

Inclusion criteria: (1) age ≥ 60 years; (2) no ethnic and geographical restrictions; (3) HRQoL score measured by EU-5Q is the outcome evaluation indicator; (4) RCT, case–control trials, high-quality original literature of cohort studies and cross-sectional survey.

Exclusion criteria: (1) animal experiments, uncontrolled trials, case reports, and reviews; (2) the inclusion and exclusion criteria for the study were not clear or reasonable; (3) the full text could not be obtained or the original data were incomplete.

### Search strategy

**Figure 1:**
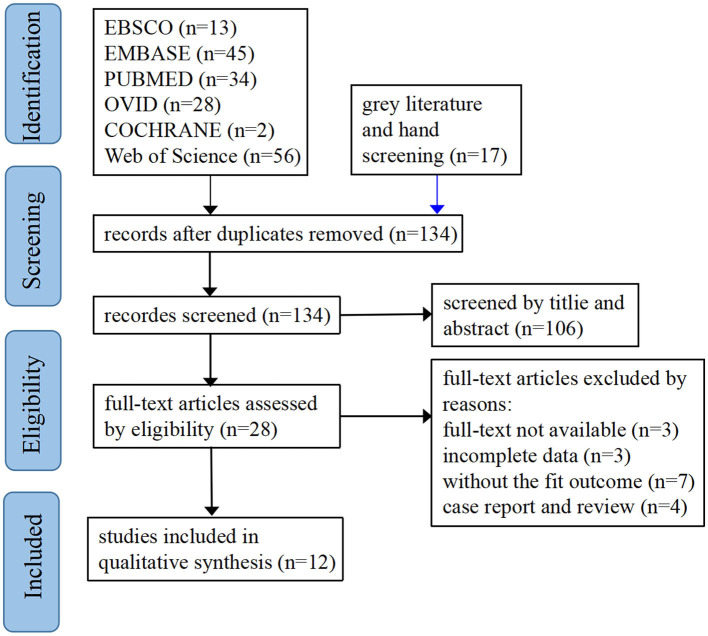
PRISMA flow diagram of screening studies process.

We searched online medical databases, including Ebsco, Embase, PubMed, Ovid, Cochrane Library, and Web of Science databases. We built a study library through screening clinical trials and original data research that met the inclusion and exclusion criteria. The databases were established until 30 November 2022. We used the following keywords: geriatric, elderly, femoral neck fractures, hip, joint replacement, total hip replacement, hemi-hip replacement, femoral head replacement, total hip arthroplasty, hemiarthroplasty, health-related quality of life, quality of life, nursing outcomes, surgical outcomes, etc. We used MeSH Terms in the four databases EBSCO, PubMed, Cochrane Library and Web of Science. All searches used the PICO principle under evidence-based nursing. This method used a broad search strategy: (elderly AND femoral neck fracture^*^ AND (“total hip arthroplasty” OR hemiarthroplasty) AND “health related quality of life”). We performed a snowball search of the included studies according to the PRISMA guidelines and the screening studies process is shown in Figure 1.

### Study selection

We got 178 pieces of literature. This was achieved by screening the article title and abstract and through full-text reading. Finally, 12 articles fit the theme. There were four RCTs, three cross-sectional studies, two cohort studies, and three case–control trials.

### Characteristics of the included studies

The baseline data of the 12 included studies are shown in Table 1. They include four comparisons of HRQoL in elderly patients after THA and hemiarthroplasty surgery (11, 13, 17, 18), and the others were on the assessment of HRQoL after hemiarthroplasty in elderly patients with different interventions (1–4, 12, 14–16). We analyzed two of the four RCT studies to compare the scores of HRQoL between elderly patients with THA and patients with hemiarthroplasty (Figure 4) (11, 13). The other two RCT studies are about the outcomes of hemiarthroplasty under different interventions, and we created subgroups to analyze the HRQoL scores (Figure 5A) (2, 12). A subgroup of cross-sectional surveys is shown in Figure 5B (3, 18), a subgroup of case–control trials is shown in Figure 5C (1, 14), and a subgroup of cohort studies is shown in Figure 5D (15, 16).

**Table 1:** Baseline data and quality evaluation of the 12 included articles.

**Author Year**	**Country**	**Study type**	**Patients number**	**Survey**	**Follow-up**	**Intervention**	**Case and control**
Hedbeck et al. (11)	Sweden	RCT	120	/	1, 2, 4years	/	THA vs. Hemiarthroplasty
Hedbeck et al. (12)	Sweden	RCT	120	/	4, 12months	/	Unipolar Hemiarthroplasty vs. Bipolar Hemiarthroplasty
Sköldenberg et al. (13)	Sweden	RCT	120	/	3months, 1, 2, 4years	/	THA vs. Hemiarthroplasty
Solarino et al. (1)	Italy	CC	118	January 2016 to June 2017	1, 3, 6months, 1, 2years	Molecular neck	Senior orthopedic surgeons vs. Orthopedic residents
Tian et al. (14)	China	CC	92	July to December, 2018	1, 3months	ERAS	Hemiarthroplasty
Wang et al. (4)	China	CC	226	2015 to 2019	24~78months	/	THA vs. Hemiarthroplasty with neuromuscular imbalance
Chammout et al. (15)	Sweden	CS	98	/	3months, 1year	/	Primary Hemiarthroplasty with cognitive dysfunction
Sebastian et al. (16)	Sweden	CS	188	February 2012 to July 2014	1year	/	Hemiarthroplasty vs. cognitive impairment Hemiarthroplasty
Pass et al. (3)	Germany	CSS	4662	2016 to 2018	7, 120days	/	THA vs. Hemiarthroplasty
Gjertsen et al. (17)	Norway	CSS	10325	2005 to 2012	4, 12months	/	THA vs. Hemiarthroplasty
Leonardsson et al. (18)	Sweden	CSS	5902	August 15 to October 25, 2010	7~24months	/	THA vs. Hemiarthroplasty
Fernandez et al. (2)	England	RCT	1225	/	4months	/	Cemented vs. uncemented Hemiarthroplasty

RCT, randomized controlled trial; CC, case control trial; CS, cohort study; CSS, cross-sectional study; THA, total hip arthroplasty; ERAS, enhanced recovery after surgery.

### Data extraction and evaluation of evidence

Two researchers independently evaluated the included 12 articles, and they discussed or asked a third senior professional expert to give a suggestion when there was any doubt about the quality evaluation. Data from the included literature were organized and entered into the software RevMan 5.4.1 by two independent researchers.

The cross-sectional survey was evaluated using the American Health Care Quality and Research Institutions Evaluation Tool, the quality of case–control and cohort studies was evaluated by the Newcastle–Ottawa evaluation tool, and the quality evaluation of RCTs was performed by the risk of bias assessment tool from the Cochrane Library.

### Outcome measures

The health-related quality of life (HRQoL) scale is assessed using the EU-5Q scale, and this includes five dimensions, namely, mobility, self-care ability, daily activities (such as work, study, housework, and leisure activities), pain or discomfort, and anxiety or depression. The data that we collected included dichotomous variables from the five EU-5Q dimensions as well as continuous variables for the mean and standard deviation of HRQoL and interquartile range for the overall patient population. We aggregated and analyzed different types of variables separately. Furthermore, it also extracted monitoring data on nursing outcomes and rehabilitation effects, and the EU-5Q scale includes both physiological structure and functional testing evaluation. Items can also effectively assess the mental health status of elderly patients, which is helpful for us to identify the problem and then treat it symptomatically. The HRQoL scores of patients after 1 month of prognosis were significantly higher than pre-operation, but they began to decline at 12 months after operation (11, 12, 16). Therefore, more problems are reported after intertrochanteric fractures than basal-type fractures and intracapsular femoral fractures, it is a key issue for surgeons to carefully consider the choice of prosthesis type (17).

### Statistical analysis

The Mantel–Haenszel model and odds ratios (ORs) with 95% confidence intervals (95% CIs) for outcomes were used to compare dichotomous variables. Mean and standard deviation were used for analysis when the units of continuous variables were consistent. A *P* < 0.05 was considered statistically significant. Statistical heterogeneity between trials was evaluated using the I^2^ test. Heterogeneity was considered when I^2^ > 50% and *P* ≤ 0.100. Then, the source of heterogeneity was analyzed, and the random effects model was used for meta-analysis. When I^2^ ≤ 50% and *P* > 0.100, there was no heterogeneity, and the fixed effects model was used for meta-analysis. The aforementioned statistical values were calculated using the statistical method provided by the RevMan 5.4.1 software.

### Risk of bias in the included studies

**Figure 2:**
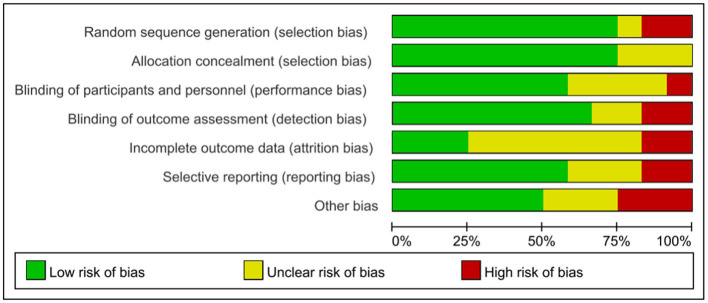
Risk of bias graph of included studies.

**Figure 3:**
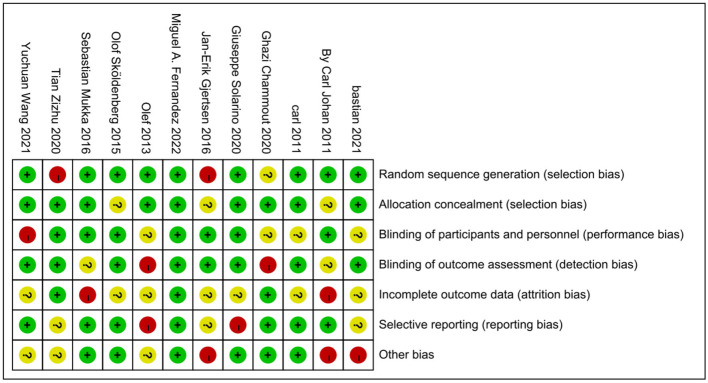
Risk of bias summary of included studies.

Random sequence generation, allocation concealment, blinding of participants and personnel, blinding of outcome assessment, incomplete outcome data, and selective reporting were evaluated as the study bias according to the Cochrane Handbook of Systematic Review (19). A risk-of-bias graph and risk-of-bias summary generated by RevMan 5.4.1 software are shown in Figures 2, 3, respectively.

## Meta-analysis outcomes

### Scores of EU-5Q between hip arthroplasty

**Figure 4:**
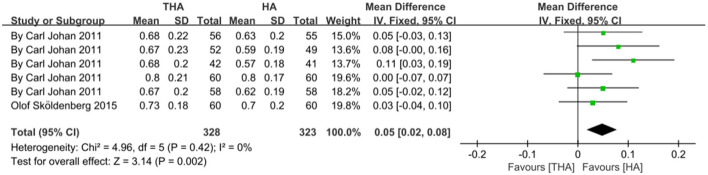
Forest plot depicting the relationship of health-related quality of life between THA and HA.

There are two types of RCT literature on the comparison of HRQoL assessment after THA and hemiarthroplasty in elderly patients. The HRQoL scores of THA are higher than hemiarthroplasty. THA is the preferred surgical method for elderly patients with FNFs, its mobility and self-care ability, as well as psychological status are better than hemiarthroplasty surgery. The outcomes are shown in Figure 4, which is consistent with the result of another case–control study comparing THA and hemiarthroplasty (4).

### Scores of EU-5Q under hemiarthroplasty

**Figure 5:**
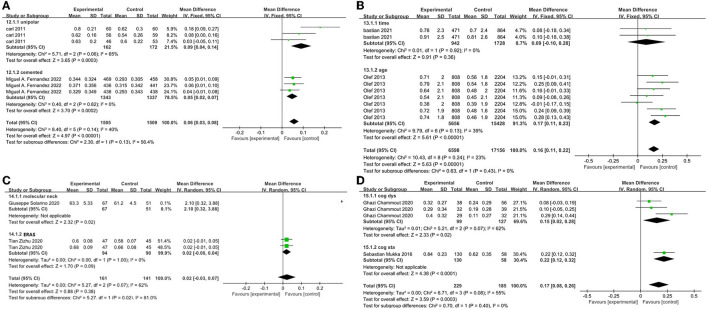
**(A–D)** Forest plot depicting.

The EU-5Q scores improved the HRQoL of hemiarthroplasty surgery in elderly patients, such as ERAS concept, neurocognitive, and different prosthesis-type interventions. As shown in Figure 5, we performed a meta-integration by literature study type and performed a subgroup analysis under different interventions. Hedbeck published an RCT of a unipolar/bipolar femoral head and Fernandez published an RCT of cemented/uncemented prostheses in elderly patients (2, 11). After integrating these two studies, we found that the HRQoL score after interventions was significantly superior to the control group (*P* < 0.01). Figure 5A shows the OR value is 0.06, 95% CI [0.03, 0.08]. This demonstrates that the EU-5Q scale could assess differences in HRQoL (12, 20). The pass has performed a retrospective analysis from the Geriatric Trauma Center Registry, there was no statistically significant difference in HRQoL scores between 1 week and 3 months after surgery, but clinical scores increased with time, and it is certain that the 3-month HRQoL average level was >1 week (Figure 5B) (3). Similarly, Tian performed the concept of ERAS on elderly patients with hemiarthroplasty. After 1 and 3 months of follow-up times, the HRQoL scores increased, but the difference between the two time periods was not statistically significant (Figure 5C) (14). However, in Chammout's prospective cohort study, there were statistically significant differences (*P* = 0.02) in HRQoL scores at 3 and 12 months after surgery (Figure 5D) (15). As shown in Figure 5B, Leonardsson followed 14 months of THA and hemiarthroplasty surgery for displaced FNFs in different age groups in elderly patients, and HRQoL scores were statistically significant (*P* < 0.001) (18). In a word, the HRQoL score after hip arthroplasty for elderly patients with FNFs should have shown rapid recovery in the first 6 months or first year, during which time the incidence of complications is higher (21).

### Publication bias

Solarino evaluated HRQoL in the elderly after 2 years, and the outcomes were not statistically significant, possibly owing to their telephone interview methods. The online telephone method perhaps neglected some important indicators, especially the patient's neurocognitive status motoring self-control and the ability to cooperate in physiotherapy treatment (1). The HRQoL scores of the observation group were dominant, and the results were statistically different. Results from both cohort studies and RCT studies were statistically significant. The bias published in this meta-analysis can be ignored. Moreover, the differences in HRQoL scores after intervention need to be verified in a multi-center, large-sample RCT that can refer to this research design (2).

## Discussion

Studies have shown that the postoperative outcomes of elderly patients with hip arthroplasty are affected by a variety of factors, the physical quality of patients, and rehabilitation nursing measures are positively correlated with the prognosis of patients (22). They assessed prognosis status by the EU-5Q (22).

### Influencing factors of medical level

The development of the medical level has promoted the improvement of medical quality. Analyzing these studies is the key to improving the hip arthroplasty quality of elderly patients and is also an important part of patients' surgical expectations. Although HRQoL in THA has obvious advantages, it still has obvious defects, which are worthy of further in-depth discussion and scientific and reasonable clinical research in the future (23, 24). Perhaps it is inconclusive about the best surgical method for elderly patients with FNFs. This is an effective surgical program that needs to consider comprehensively the patients' basic physical conditions (comorbidity index, frailty index, etc.) to choose the optimal treatment and nursing measures. Modular surgical training residents can help them efficiently restore femoral offset and balance damaged soft tissue, reducing the incidence of postoperative abductor imbalance, hip pain, and acetabular wear (1). Its dislocation rate of THA is higher than hemiarthroplasty and internal fixation; however, there is still controversy about the dislocation rate of THA in elderly patients (25, 26). Although unstable elderly patients with FNFs are mostly treated with THA surgery, internal fixation, hemiarthroplasty surgery are recommended for stable FNFs (27).

Wang found that THA was associated with better functional outcomes, quality of life, and lower pain intensities, despite longer operation times and higher intraoperative blood loss (4). This is consistent with the findings of Yu et al. (25) and Hopley et al. (26). The evaluation of HRQoL in elderly patients with FNFs is associated with the outcome of THA and hemiarthroplasty operation. Younger orthopedic residents should gain more experience working with elderly patients with FNFs under supervision from senior orthopedic surgeons to acquire proficient skills for hip arthroplasty operations (1). Simultaneously, orthopedic nurses need continuing education about hip arthroplasty perioperative management. The first thing that orthopedic surgeons think about is the surgical effect of THA and hemiarthroplasty, such as the functional recovery benefits and pain alleviation after the surgery (28). THA surgery has obvious advantages in terms of prognosis. What constitutes the optimal surgical program for elderly FNFs has been controversial (23). We do not dismiss that hemiarthroplasty is generally recognized by physicians and patients, and we have thus integrated studies on HRQoL with hemiarthroplasty in elderly patients with FNFs and found favorable and unfavorable factors affecting patients' recovery. One study of a 4-year follow-up between THA and hemiarthroplasty showed significant differences in HRQoL scores, measured by EU-5Q, with THA superior to hemiarthroplasty after 48 months (*P* < 0.039) (11). The findings demonstrated that rapid rehabilitation interventions, modular training of residents, and choice of prosthesis type all contribute to improving HRQoL scores after hemiarthroplasty in elderly patients, both in medical education and in future clinical practice. The improvement of the management concept is effective for elderly surgical rehabilitation.

### Influencing factors from patients

As we age, various physiological functions of the human body decline, and this is closely related to the rehabilitation effect (18). However, HRQoL scores of elderly patients with hip arthroplasty are statistically different with age, and there is a clinically significant difference in the prognosis after 1 year of follow-up. Therefore, age is an independent risk factor affecting the surgical effect. Moreover, the most important thing is that clinicians should not only use age as an important reference for performing surgical programs but also need to consider physiological factors (29). There are significant statistical differences in the HRQoL scores, such as drawing the line between 70 and 80 years (17). The funnel plot shows the heterogeneity of retrospective case–control trials of neuromuscular imbalances, suggesting that elderly THA with neuromuscular disease were also reported to be superior to a hemiarthroplasty, they were superior to patient rehabilitation in reasons of EU-5Q evaluation, hemiarthroplasty is a safe and available treatment option for elderly patients with FNFs, but its clinical outcomes are a little poor (risk of dislocation and prosthetic fracture), and it is urgent to explore the optimal rehabilitation care plan for such disorders (23). As is well known, age was an important factor influencing the recovery of HRQoL after hip arthroplasty with FNFs in elderly patients, mobility recovery becomes slower and slower with aging. Supplementally, the ERAS nursing intervention improved HRQoL scores after hemiarthroplasty surgery in elderly patients with FNFs. In the perioperative nursing process, we can integrate systematic nursing routines or emergency plans to improve the elderly hip arthroplasty quality.

Neurocognitive function during the perioperative period is highly related to surgery outcomes, surgeons should take into account the elderly patients' cognitive function whose risk factors may refer to neurocognitive complications, such as perioperative neurocognitive disorders (30, 31). Thus, further study is needed on the subject of the correlation between elderly cognitive function and perioperative medical quality; for example, there was a study indicating that neuropsychiatric disease occurred subsequently after cobalt and chromium metallosis following metal-on-metal implant failure (32). It is recommended to carry out future follow-up trials of more than half a year can identify the main clinical factors that affect the recovery of HRQoL (15).

### Rehabilitation factors

The success of hip arthroplasty in elderly patients is the first key link with patients' rehabilitation. Postoperative rehabilitation and nursing measures are the most important factors. Moreover, HRQoL scores after hip arthroplasty for elderly patients with FNFs have accelerated recovery in the first 6 months (21), and the dislocation rate is 2.7 times higher in the first year of follow-up time (24). For elderly patients with hip arthroplasty, it is relatively difficult to implement postoperative nursing interventions and promote the rehabilitation process due to the existence of objective factors such as hearing loss, poor understanding ability, and poor compliance (33). The medical staff needs more patience, carefulness, love, and responsibility to provide medical services for elderly patients with hip arthroplasty. Preoperative preparation, intraoperative care, and postoperative observation are the three most critical stages for elderly patients with hip arthroplasty.

Enhanced recovery after surgery (ERAS) nursing speeds up the rehabilitation process, shortens the hospital stay time, reduces hospitalization cost, improves patient satisfaction, and increases the operation rate within 48 h. In addition, the HRQoL score shows a statistically significant difference, and the EU-5Q scale is a promoting factor for the prognosis (14). The utility scores on the EU-5Q questionnaire in the THA group, bipolar hemiarthroplasty group, cemented group, and senior surgeons group would be modestly but significantly better than the control group in these included studies. The THA surgery plan, type of bipolar prosthesis, type cemented, and highly qualified surgeons are protective factors for elderly FNFs surgery. All of the above provides a scientific basis for the FNFs treatment. Neuromuscular imbalance (stroke, Parkinson's disease, etc.) and cognitive impairment are impediments to FNFs surgery in elderly patients, and patients have poor postoperative HRQoL scores, but hemiarthroplasty surgery can be considered a safer option. In view of the low OR value between the THA and hemiarthroplasty groups, in addition to the EU-5Q questionnaire, the five dimensions of the scale can be evaluated separately, both of which can sensitively prove the advantage of surgical outcome variables (17).

## Conclusion

In this study, although there is more prior evidence about THA surgery, hemiarthroplasty also has good functional rehabilitation. Consequently, this points to new directions for the treatment and nursing of elderly femoral neck fractures with cognitive or mobility impairment. In the future, the correlation between surgical treatment outcomes and preoperative and postoperative neurocognitive degradation in elderly patients with FNFs should be further investigated.

The findings demonstrated that hip arthroplasty intervention should be implemented in 6 months because there is a significant statistical difference in the HRQoL score during this period. Moreover, it is effective for rapid elderly surgical rehabilitation including modular training of residents, prosthesis type, and improvement of management concept.

### Limitation

The influence of the patient's neurocognitive status in physiotherapy treatment should be deepened. Most important of all, the study of the correlation between surgical treatment outcomes and preoperative and postoperative neurocognitive degradation in elderly patients should be further investigated. Moreover, elderly people with obesity after hip surgery, the mood variables, the society supporting system, etc. all should be associated with exploring its effects after surgery, and the beneficial measurement questionnaire can support effective interventions for elderly patients with hip arthroplasty.

## Data availability statement

The original contributions presented in the study are included in the article/supplementary material, further inquiries can be directed to the corresponding author.

## Author contributions

RL conceived and designed the framework of the article. YS and XR searched and screened the literature, evaluated the quality of the included articles, completed the manuscript with the help of RL, and consulted YW and LH when there was doubt. All authors contributed to the article and approved the submitted version.
